# Lanthanide-doped nanoscintillators

**DOI:** 10.1038/s41377-022-00987-2

**Published:** 2022-09-29

**Authors:** Jean-Claude Georges Bünzli

**Affiliations:** 1 Institute of Chemical Sciences and Engineering, Swiss Federal Institute of Technology, Lausanne (EPFL), Lausanne, Switzerland; 2 Department of Biomedical Engineering, Southern University of Science and Technology (SUSTech), Shenzhen, China

**Keywords:** Optics and photonics, Optical materials and structures

## Abstract

Lanthanide-doped nanoscintillators are taking the lead in several important fields including radiation detection, biomedicine, both at the level of diagnosis and therapy, and information encoding.

A scintillator is a material exhibiting luminescence when excited by ionizing radiations such as X-rays, γ-rays, or radioactive particles (α, β, neutrons). During their passage through the scintillator material, ionizing radiations lose energy, part of which is transformed into photons which are then counted by a photodetector^1^.

An obvious application of scintillators is the spotting and quantitation of ionizing radiations, as was done as early as 1903 by Sir William Crookes for α particles, with a zinc sulfide screen and naked eye detection. Other applications include nuclear fuel and waste monitoring, medical diagnosis, mainly computed tomography, positron emission tomography, and X-ray excited optical imaging, as well as nuclear medicine. A broad variety of materials exhibit scintillation, ranging from noble gases to organic molecules, under the form or crystals, solutions or doped into a polymeric material, and to purely inorganic compounds such as alkali metal halides, sodium and cesium iodides doped with thallium being archetypical examples. Specific characteristics of a scintillator include its scintillation efficiency, light yield, defined as the number of emitted photons by unit energy dissipated in the scintillator by the ionizing particles (photons/MeV), response time, preferably in the ns range, energy and spatial resolution, linearity of its response with the incident radiation energy, and stability. Furthermore, a good spectral match between the emission range of the scintillator and the photodetector response is instrumental to high sensitivity (Fig. 1).

**Fig. 1:**
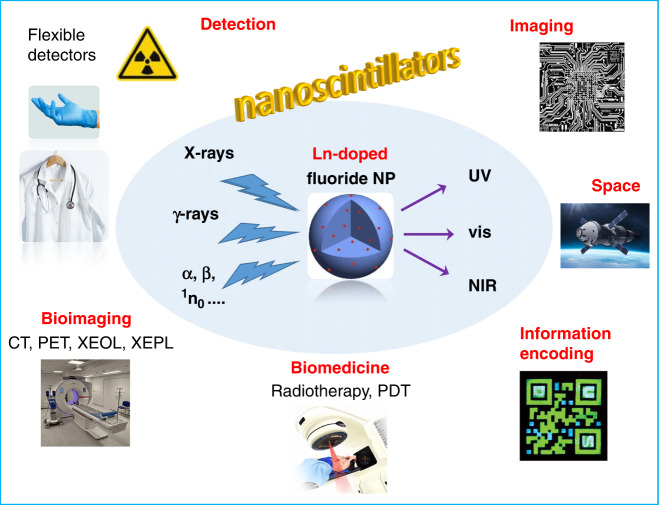
Main applications of nanoscintillators. Key. NP nanoparticle, CT computed tomography, PET positron-emission tomography, XEOL X-ray excited optical luminescence, XEPL X-ray excited persistent luminescence, PDT photodynamic therapy

Inorganic single-crystal scintillators have generally high light yield and large energy resolution. However, they are difficult to synthesize and their emission wavelength cannot be tuned. To improve this situation, researchers recently turned to metal halide perovskites^2,3^, including their nanoscale declination^4^, that demonstrated adequate wavelength tunability and photophysical properties. These materials represent a welcome advance in the field but as far as medical imaging is concerned, a crucial point is not addressed, namely the need for performing the imaging with deep-tissue penetrating near-infrared light. Here come lanthanides into play. Indeed, these metal ions are luminescent with narrow emission bands spanning the UV-visible-NIR range up to about 3 μm^5^. They can easily be incorporated into a wealth of inorganic matrices, including nanoparticles and their luminescence can be X-ray excited^6^. Moreover, their similar chemical properties make it easy to tune the emission of a given material by doping specific lanthanide ions, or a mixture of them, into it.

A recent review article published in eLight, sister journal of Light: Science & Applications, written by Professor Paras N. Prasad from State University of New York, Professor Xu Shiqing and Associate Professor Lei Lei from China Jiliang University, and their collaborators, describes the world of lanthanide-doped nanoscintillators and photon converters^7^. The review essentially focuses on lanthanide fluoride nanoparticles doped with various emissive lanthanide ions. The corresponding nanoscintillators are easy and cheap to process and these materials help solving important problems in medical imaging. One has been mentioned above, replacing visible-emitting probes with NIR-emitting ones, particularly in the NIR-II biological window (1000–1700 nm). Another one pertains to long-persistent luminescence bioprobes that are useful for following reactions in cells and, also, for monitoring the advancement of medical therapies^8^. Yet, most of these probes have to be charged by UV-light before being introduced into the biological medium or the investigated tissue. In this case, even if the probe emits in the NIR-II range, recharging it in vivo is difficult due to the low penetration of UV light. On the other hand, X-rays penetrate deeply into tissues and are therefore ideal for recharging these NIR-II emitting nanoprobes.

Additionally, nanoscintillators can be introduced into biocompatible polymers such as polydimethylsiloxane (PDMS) yielding flexible detectors that are convenient for the imaging of curved 3D objects, a difficult, or even impossible, task with flat-panel X-ray detectors^9^. A main focus of the review is therefore on X-ray excited optical imaging (XEOL) and X-ray excited persistent luminescence (XEPL).

The authors devote a large section of their manuscript to discussing the mechanisms involved in X-ray to UV, visible, and NIR conversion, as well as those pertaining to the generation of persistent luminescence, making the review an excellent tutorial for scientists entering the field. Convincing examples of XEOL/XEPL imaging follow suit, including circuit boards, biological material, and various hidden or encapsulated objects. Comparison between flat-panel detectors and XEPL detection with flexible polymers (sometimes referred to as X-ray luminescence extension imaging, Xr-LEI^9^) is particularly convincing. A good share of examples are in the field of biomedicine, with multimodal imaging, radiotherapy monitoring, X-ray enabled photodynamic treatment, to name but a few. Another important field of application lies in information encoding in luminescent QR codes or for multidimensional information storage.

However, all problems associated with the design of high-performance lanthanide-doped nanoscintillators are not completely solved. In particular combining bright XEOL intensity with fast decay rate is not granted and optimization of the composition and structure of nanoparticles incorporating several lanthanide ions for broadband detection still needs dedicated efforts. These aspects are presently the subject of intensive studies and further achievements are on their way. Therefore, perspectives for lanthanide-doped nanoscintillators are bright. Their ease of synthesis and stability are considerable assets and they definitively enable improving several aspects of crucial applications in industrial imaging, information storage, and biomedicine. Regarding the latter, X-ray excited luminescence is starting to overtake traditional luminescence optical imaging. Radiation detection is also vital for space applications and for all professionals dealing with radioactive materials. In particular, flexible detectors incorporated into gloves or textiles (lab coats) could help determining not only the received irradiation dose, but, also, its precise cartography. Moreover, the ability of 3D imaging could be exploited in diagnosis, for instance in breast mammography.
